# Strong linkage between parrotfish functions and habitat characteristics

**DOI:** 10.1371/journal.pone.0315179

**Published:** 2024-12-11

**Authors:** Ángela Randazzo-Eisemann, Ana Lilia Molina-Hernández, Lorenzo Alvarez-Filip, Joaquín Rodrigo Garza-Pérez

**Affiliations:** 1 Unidad Académica de Sistemas Arrecifales, Instituto de Ciencias del Mar y Limnología, Universidad Nacional Autónoma de México, Puerto Morelos, Quintana Roo, México; 2 Posgrado en Ciencias del Mar y Limnología, Universidad Nacional Autónoma de México, Ciudad de México, México; 3 PIESACOM, UMDI-Sisal, Facultad de Ciencias, Universidad Nacional Autónoma de México, Sisal, México; University of Mauritius, MAURITIUS

## Abstract

Phase shifts from hard coral to macroalgae have led to the formulation of a top-down herbivory paradigm, whose assumption is that a reduction in herbivory is the primary driver of these changes. Caribbean parrotfish from *Scarus* and *Sparisoma* genera are usually known as main reef herbivorous. Yet, they are a diverse group of organisms that perform multiple functions, including the bioerosion of reef structures. Generalizing functions at the group level likely explains why the direct effects of parrotfish on macroalgae regulation are not always evident. In this study, we tested the hypothesis that parrotfish potential functions are strongly linked to the habitat’s benthic characteristics. Furthermore, we expect that the parrotfish bioerosion potential will be highly sensitive to changes in benthic conditions, while herbivory will be more robust. We conducted *in situ* benthic and parrotfish surveys across the diverse reefscape of the remote Alacranes Reef, the most extensive system in the Gulf of Mexico. Both bioerosion and herbivory potential were highest in the most complex and structured sites, while only macroalgae removal was high in deep low-coral cover sites dominated by fleshy macroalgae. Interestingly, both functions were highly diminished in shallow and reticulated inner reefs dominated by turf algae and cyanobacteria, suggesting that even the herbivory function can be depleted under unfavorable benthic conditions. Our findings highlight the need to reconsider parrotfish management strategies to account for the specific roles of different species and consider reciprocal benthic-fish interactions.

## 1. Introduction

The loss of coral reef resilience in the Caribbean has been largely described as the phase shift from hard coral to macroalgae loss [1, 2]. This approach led to the formulation of a top-down herbivory paradigm, which posits that a decrease in herbivory -caused by chronic parrotfish overfishing in the Caribbean and the mass mortality of *Diadema antillarum* in the eighties- induced unchecked increases in macroalgae cover, which can outcompete corals for space and impair the maintenance and recovery of coral assemblages by increasing pathogen loads, reducing growth, fecundity, and recruitment [3, 4]. Herbivore management, mainly promoted through the protection of parrotfishes, is thus considered a leading tool for resilience-based approaches that policy-makers and managers can apply at operational and ecologically relevant scales in the Caribbean region [5, 6].

Certainly, parrotfishes play critical functional roles in structuring coral reef habitats as key herbivores that facilitate the maintenance and recovery of coral-dominated reefs by controlling algae and provisioning space for the recruitment of corals [7]. However, the relationship between fishes and benthic organisms is complex, reciprocal, and operates on multiple scales [5], so the direct effects of herbivorous fishes on macroalgae are not evident in all cases. For example, several studies in the Caribbean have reported no direct relationship between macroalgae cover and herbivorous fish abundance, whether they consider the number of individuals or their biomass [8, 9]. This inconsistency highlights the need to integrate other metrics that allow us to more robustly estimate and describe the influence of parrotfish as herbivores on benthic communities [10].

Besides, despite apparent functional redundancy, large functional differences among parrotfish species are driven by differences in diet and other attributes, including the habitats they frequented, the types of substrates they fed from, and the spatial scale at which they foraged [11], indicating that parrotfish species can play varying roles in coral ecosystems [10]. For instance, Caribbean parrotfish are mainly associated with their herbivory potential, while some Caribbean parrotfish species also play an important role in bioerosion, a core reef process [12, 13]. These different roles highlight the importance of enhancing the traditional parrotfish management paradigm, which assumes a functional equivalency among different parrotfish species, by integrating specific functional variability [10].

Furthermore, functional variability in Caribbean parrotfishes is also size-dependent [10], and recent studies have highlighted that large scarids have higher bioerosion potential, whereas medium-sized species have higher herbivory potential [10]. Additionally, it has been demonstrated that benthic habitat degradation and the consequent loss of architectural complexity and coral cover [14] negatively impact populations of large-bodied parrotfish due to reproductive traits and habitat dependency [15–22]. Based on this pattern, we might expect that habitat characteristics influence parrotfish assemblages and their functions at the reef scale. Consequently, parrotfish bioerosion may be more sensitive to changes in parrotfish assemblages than herbivory [10, 23].

**Fig 1:**
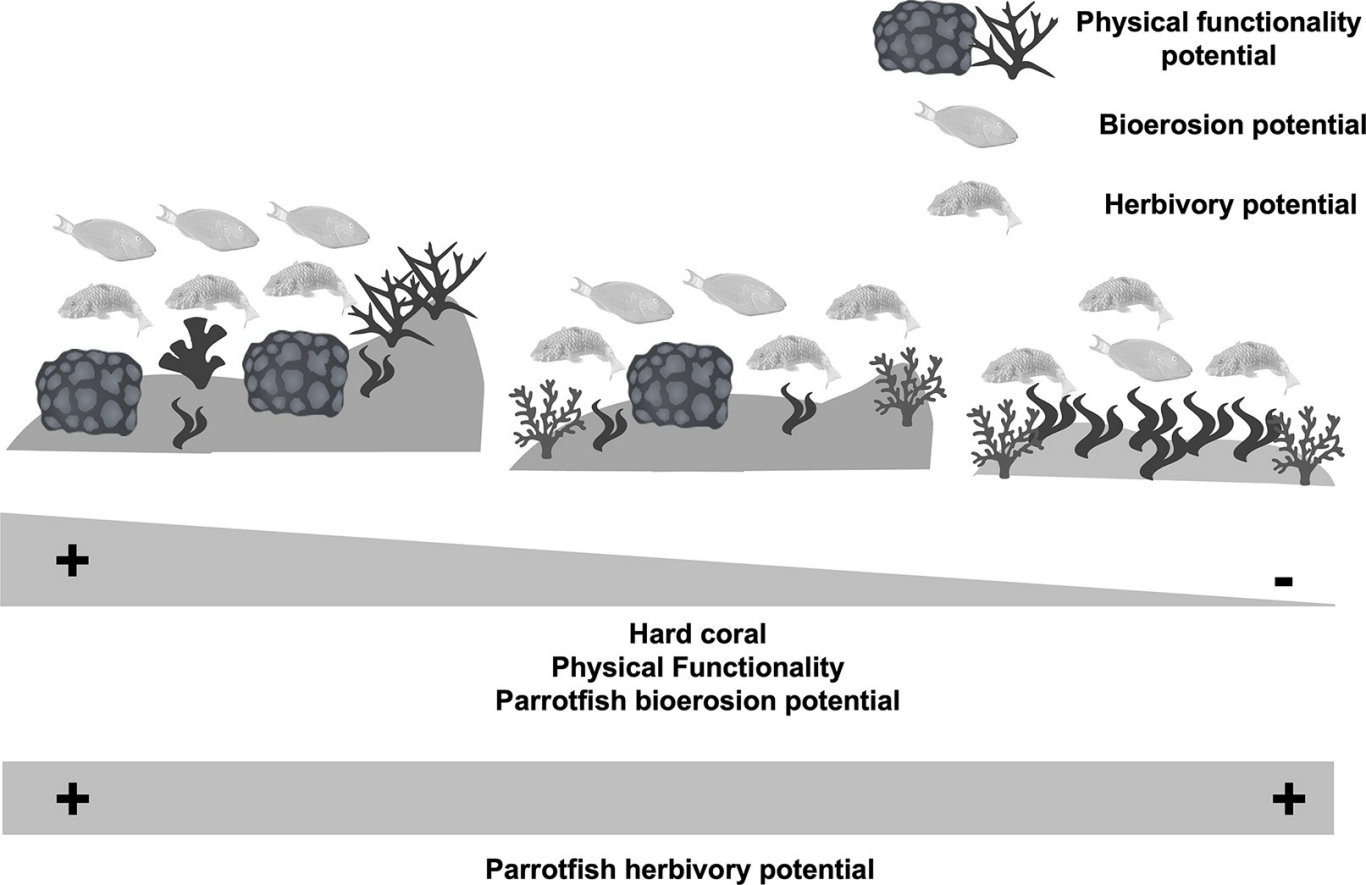
**This study hypothesizes that we would expect greater parrotfish bioerosion potential at sites with higher live coral cover and the reef’s physical functionality.** We also expect that the herbivory potential is a more robust function despite the different benthic habitat conditions.

Yet, we still have little understanding of how changes in parrotfish assemblages and their associated potential functions of bioerosion and macroalgae consumption respond to different benthic conditions (i.e., coral-dominated vs. macroalgae-dominated) and reef habitats [23]. In this study, we tested the hypothesis that parrotfish functions are strongly linked to the habitat’s benthic characteristics. Considering that the species with the greatest potential for regulating macroalgae are more generalist species, while highly bioeroding species are more sensitive to habitat conditions [10, 16], we expect that the bioerosion potential of parrotfish will vary according to the gradient of benthic conditions and that herbivory will be more stable despite changes in parrotfish assemblages (Fig 1).

We conducted our study across the Alacranes reef system, located in the Gulf of Mexico and part of the Wider Caribbean [24]. This remote reef system provides a meaningful baseline for descriptive comparisons of ecological processes and resilience, as the Alacranes Reef System is still driven mainly by environmental gradients rather than coral stressors [25, 26]. Coral assemblages in the Alacranes Reef are dominated by structurally complex species [25], and despite being subject to some level of fishing pressure [27, 28], the biomass of herbivores has been stable over the last decades [29]. Likewise, the system hosts a diverse and abundant parrotfish assemblage [29].

## 2. Methods

### 2.1. Study site

The Alacranes Reef is located 140 km north of the Yucatan coast in the Gulf of Mexico. It is a platform reef with a well-developed reticular network of coral patches within the reef lagoon, characterized by high geomorphological diversity and multiple reef seascapes [29, 30].

The system has a semi-circular well-developed windward reef on the eastern side, forming a continuous barrier along the north, east, and southeast. The leeward side has a less developed veneer of reef growth characterized by small patch reefs and submerged sandbars. A network of shallow reef patches gives the lagoon a reticular accretion pattern, reaching a maximum depth of 20 m in some parts of the northern and southeastern sections.

**Fig 2:**
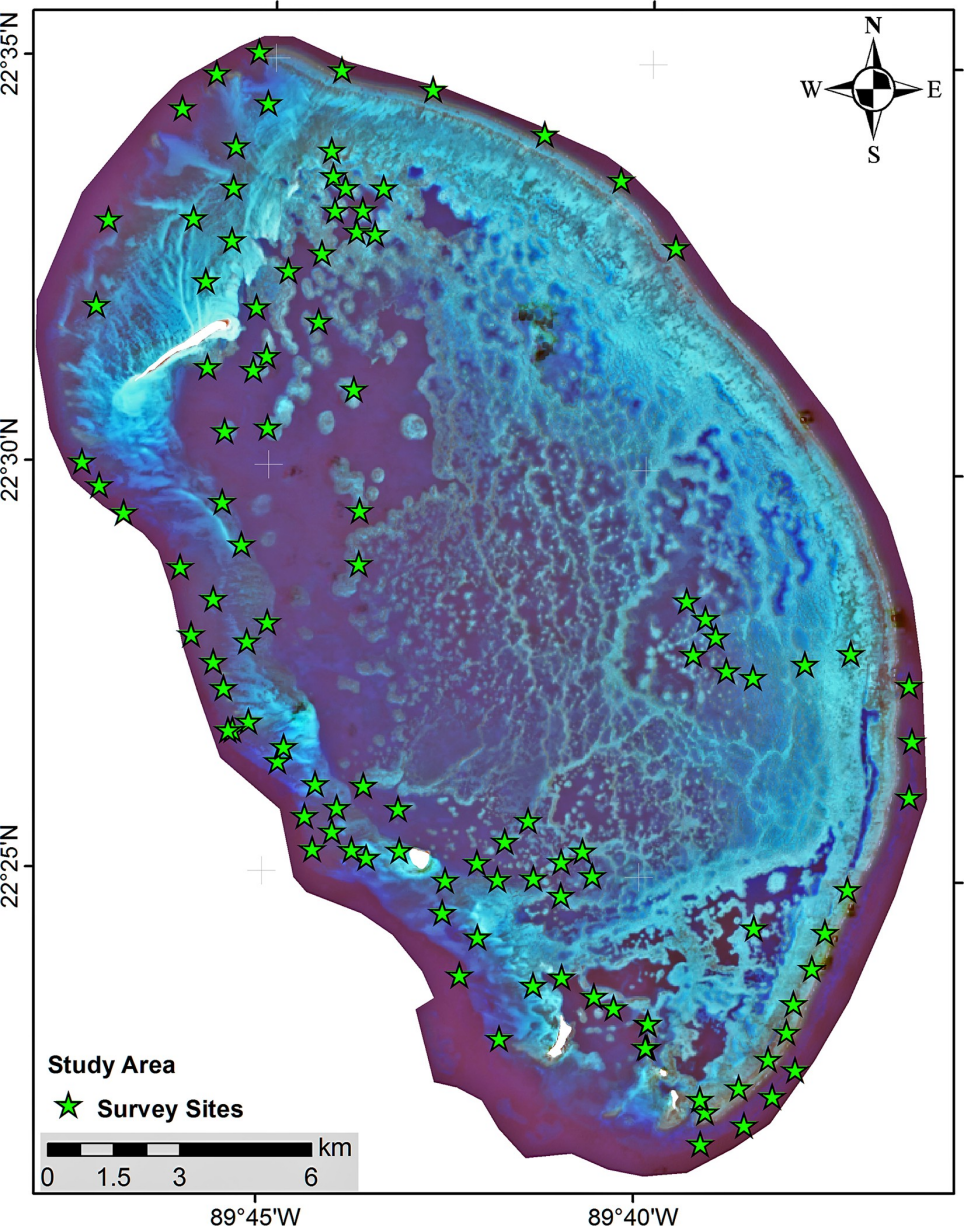
Seventy-six sites were surveyed in Alacranes Reef National Park, located in the southern Gulf of Mexico part of the Wider Caribbean. Landsat-8 image courtesy of the U.S. Geological Survey.

The sampling design has integrated seascape habitat heterogeneity by considering geomorphologic zones (fore, back, inner reef), wind exposition (windward and leeward), and bathymetry. Based on these criteria and navigation accessibility, 76 sites were selected for this study (Fig 2).

### 2.2. Data collection

Natural Protected Areas Council (CONANP), through the office managing Parque Nacional Arrecife Alacranes (PNAA), issued the authorization PO0.9DPNAA/099/2022 for park access and scientific survey activities without sample collection.

All data was collected in July and August 2022, during the rainy season. For each site, parrotfish species data (species-level identification, abundance, and total length per individual) were collected *in situ* in a non-permanent transect of 100 m^2^ (50 × 2 m) (Survey Data, S1 Dataset). Then, based on the abundance and size, the biomass of each parrotfish species per site was estimated using the allometric equation Weight = a x (TL)^b^, where a and b are fish species-specific parameters and TL the total length [31].

Reef benthic organisms were recorded along the same site transect (50 x 0.6 m) with a high-resolution camera (GoPro Hero 8 Black), maintaining the camera at a constant 0.4 m height throughout the transect for a sampling area of 30 m^2^ [32, 33]. To estimate the benthic cover percentage, 40 evenly spaced freeze-frames in each video were subsampled, and for each frame, 13 points systematically positioned on the screen were associated with a specific benthic category, such as crustose coralline algae, turf algae, turf algae with sediments, cyanobacteria, fleshy macroalgae, calcareous macroalgae, octocoral, sponge, sand, and live corals, which were identified to the taxonomic species level. For each benthic category and coral species, a percentage of the total 520 points per site was calculated (Survey Data, S1 Dataset).

Moreover, to estimate the coral species-specific functional contribution according to their capacity to construct complex three-dimensional structures, we determined the Reef Functional Index [34]. The index was calculated as follows:

$${RFI}_{S}=\frac{\sum_{i=1}^{n}{LC}_{i}*{Fc}_{i}}{100}$$ (1)

where *RFI*_*S*_ is the reef functional index at a given site *s*, *LC*_*i*_ is the cover and *Fc*_*i*_ is the functional coefficient *for a given coral species (i)*. This RFI value was transformed to the four-root value to facilitate interpretation.

Although fishing is an important additional driver mediating parrotfish benthic interactions by influencing the biology of assemblages, this key driver was not considered in this study for two reasons: (1) there is no accurate spatial data on fishing pressure at the studied sites, and (2) previous studies have found that herbivorous biomass (including parrotfish species) is stable between 1998 and 2022 [29].

### 2.3. Parrotfish functions

The parrotfish species considered for this study are common in the Wider Caribbean coral reefs: *Scarus guacamaia*, *Scarus coeruleus*, *Scarus coelestinus*, *Scarus vetula*, *Sparisoma viride*, *Sparisoma chrysopterum*, *Sparisoma rubripinne*, *Sparisoma aurofrenatum*, *Scarus taeniopterus*, and *Scarus iseri*.

We estimated the rates of bioerosion and macroalgae consumption [10] through equations proposed by former studies using census-based approaches [10, 35]. Bioerosion rates (kg CaCO3 m^-2^ yr^-1^) were calculated using the species abundance observed per area, the species-specific published bite rates (bite h^−^1), the bite volume (cm^3^), and the proportion of bites leaving scars, with all these parameters depending on the size and life phase [10, 35]. Similarly, macroalgae consumption rates (g C m^-2^ yr^-1^) were calculated using variables such as observed species abundance observed per area, as well as parameters found in literature such as the bite rate, bite area, bite volume, total algal consumption, and the proportion of macroalgae consumed [10].

**Fig 3:**
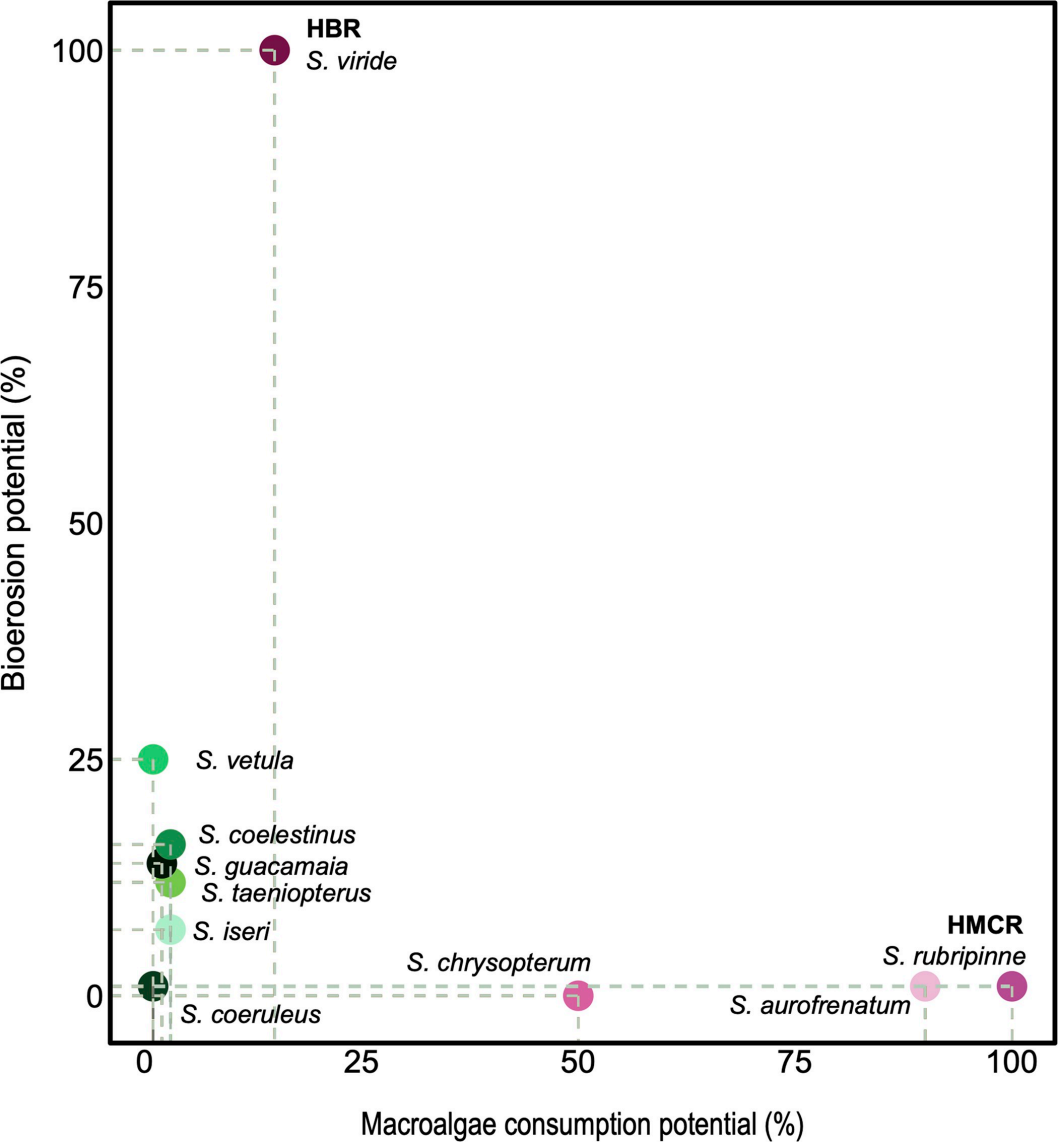
The relative potential to erode hard corals and consume macroalgae from ten main Caribbean parrotfish species for individuals of 25 cm as a reference, as all Caribbean parrotfish attained at least this size, allowing comparison between all the species studied. The highest bioerosion rate potential (HBR) was observed for *Sparisoma viride* (i.e., 40.61 kg ind^-1^ yr^-1^), and the highest macroalgae consumption rate potential (HMCR) was observed for *Sparisoma aurofrenatum* (i.e., 716.8 g C ind^-1^ yr^-1^). Based on the 100%-value for each parrotfish function, the relative potential (%) for the remaining species was estimated. Species with higher values of bioerosion are represented by pink hues, species with higher values of macroalgae consumption are represented by green hues, and species with low values of both functions are represented by turquoise.

Both parrotfish functions depend not only on the parrotfish species but also on their size and biomass [10, 35]. While the bioerosion rate is strongly driven by large species such as *Sparisoma viride*, followed to a lesser extent by *Scarus vetula*, a greater number of medium-sized species, such as *Sp*. *rubripinne*, *Sp*. *aurofrenatum*, *and Sp*. *chrysopterum* can consume relatively high amounts of macroalgae (Fig 3).

### 2.4. Ecological analyses

Based on benthic cover per site, a cluster analysis was used to split the 76 sites into groups sharing similar benthic conditions. This analysis was performed using the R functions “*kmeans*” (R base function) and “*fviz_cluster*” from the “*factoextra*” library [36]. In addition, for each group, we calculated (1) the average percentage of dominant benthic components (hard coral, octocoral, turf algae with sediments, cyanobacteria, calcareous macroalgae, and fleshy macroalgae), (2) the average coral life history strategy (*sensu* [37]), (3) the average parrotfish biomass per species, and (4) the average reef functional, erosion rate, and macroalgae consumption.

Benthic cover, parrotfish biomass per species, reef complexity, and parrotfish functions were integrated into a canonical analysis of principal coordinates (CAP) to investigate how parrotfish biomass, erosion rate, and macroalgae consumption varied among sites based on the benthic conditions of the sites and thus structural complexity (e.g., RFI). CAP performs a constrained ordination analysis in two steps [38]. In the first step, a principal coordinate analysis (PCoA) was computed using the coefficient of percentage difference (Bray‒Curtis) on the benthic cover. In the second step, a redundancy analysis (RDA) of the PCoA created above (acting as the response data) was run, constrained by the benthic groups. Furthermore, the biomass of each parrotfish species, as well as the RFI, depth, erosion rate, and macroalgae consumption, were fitted into the ordination space. This analysis was performed using the R libraries “*vegan*” (function “*capscale*” and *“envfit”*) and *“ggplot2”* [36]. Finally, we built a generalized linear model of erosion rate and macroalgae consumption as a function of benthic groups.

### 2.5. Habitat mapping

To visualize the spatial distribution of the four different habitats defined by the cluster analysis of the field sites, a habitat map of the platform was generated. Firstly, a RapidEye multispectral image from 2017 (Planet Team, 2022, Planet Application Program Interface: In Space for Life on Earth. San Francisco, CA. https://api.planet.com.) was pre-processed by applying atmospheric, radiometric, water-column corrections, a Local Sigma Filter of 9-pixel radius [39, 40], as well as masking the deep water around the reef platform.

Secondly, a supervised classification (Mahalanobis algorithm) was applied to the pre-processed image according to the benthic habitat scheme defined previously by the cluster analysis. From the 350 sites designated for this procedure (76 field sites + 274 sand and seagrass bottoms selected using expert knowledge), a subset of 262 sites were used as training fields for a five classes scheme (4 benthic groups + others such as seagrass and sand). This subset was tested for separability using the Jeffries-Matusita and Transformed Divergence indexes. The resulting classified image was post-processed using a majority analysis, and a subset of 88 sites (25% not used for the classification training) were used to assess the accuracy of the classification.

## 3. Results

**Fig 4:**
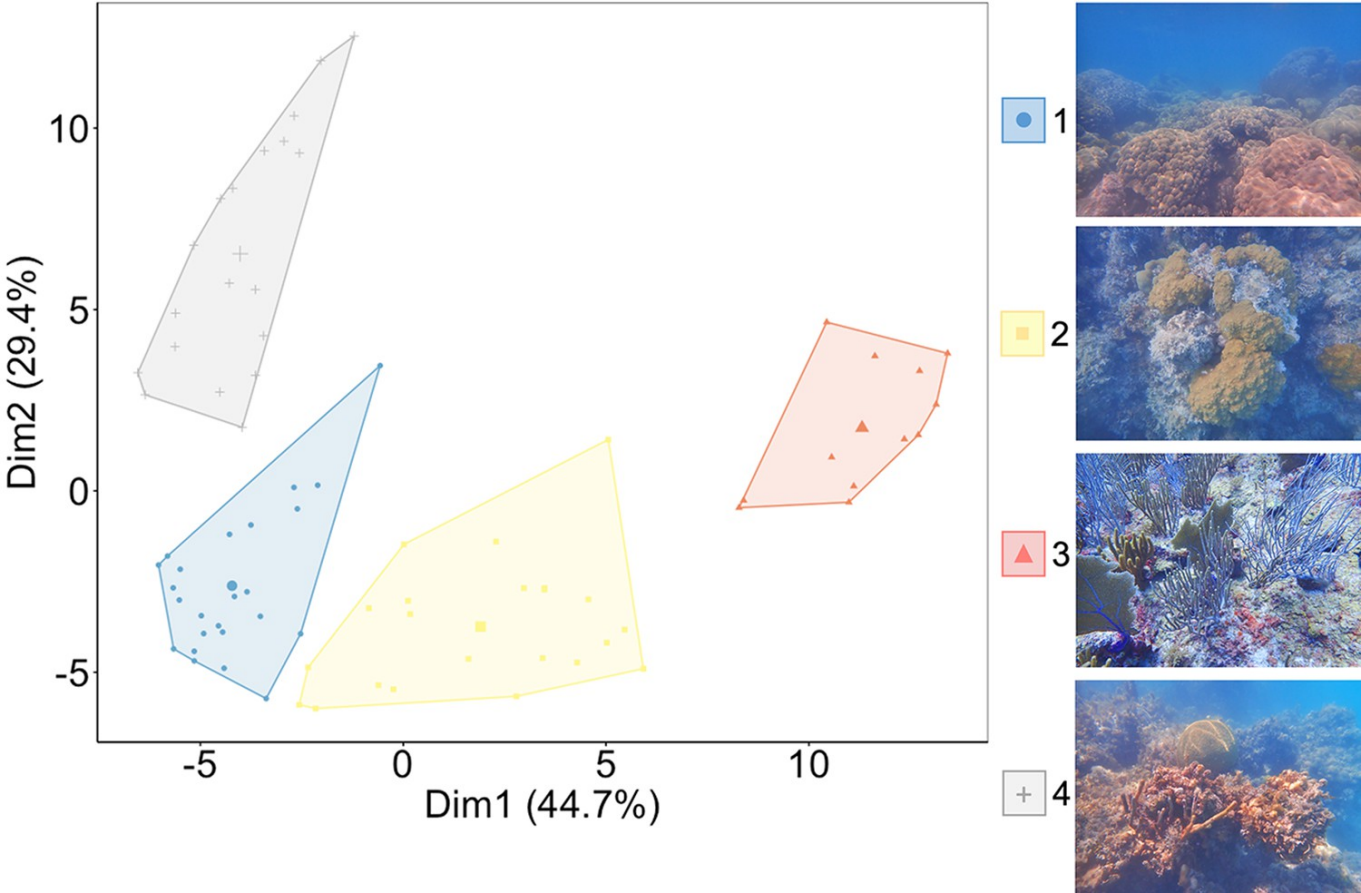
Clustering of 76 sites into four groups with different benthic conditions illustrated by seascape pictures. These two axes accounted for 74.1% of the total variability.

Based on the cluster analysis, we identified four groups of sites representing different types of habitats mainly differentiated by the cover of 1) live corals, 2) turf algae, 3) fleshy macroalgae, and 4) cyanobacteria (Fig 4 and Table 1). The sites in group 1 are shallow (2.5±0.4 m) and dominated by both hard coral and turf algae with sediments (TAS); the sites in group 2 are deeper (7.9±0.9 m) and dominated by turf algae and sediment with a similar abundance of other dominant benthic components (hard corals, fleshy macroalgae, and octocorals); the sites in group 3 are the deepest (12.1±1.5 m) and dominated by fleshy macroalgae; and the sites in group 4 are shallow (2.1±0.3 m) and dominated by cyanobacteria (Fig 4 and Table 1). Moreover, there was a simultaneous decrease in the average cover of hard coral and an increase in the average cover of fleshy macroalgae, likely due to the depth gradient from group 1 to group 3 (Table 1).

**Table 1:** Benthic conditions for each group. *LC*, *OCTO*, *TAS*, *CYAN*, *CMA*, and *FMA* are the average live coral, octocoral, and turf algae with sediment, cyanobacteria, and calcareous and fleshy macroalgae cover, respectively, and *RFI* is the reef functional index and the standard error associated with the average values.

Site Group	$\bar{Depth}(m)$	$\%\bar{LC}$	%*OCTO*	$\%\bar{TAS}$	$\%\bar{CYAN}$	$\bar{\%CMA}$	$\bar{\%FMA}$	$\bar{RFI}$	Site Number	Site %
**1**	2.5± 0.4	25±3	5±1	37±2	4±1	4±1	2±1	0.54±0.03	23	30
**2**	7.9± 0.9	13±2	16±2	23±2	4±1	2±1	15±2	0.44±0.01	22	29
**3**	12.1± 1.5	8±1	18±3	7±1	4±1	1±1	43±4	0.37±0.01	12	16
**4**	2.1± 0.3	12±1	2±1	19±2	25±3	24±3	2±1	0.45±0.01	19	25

**Fig 5:**
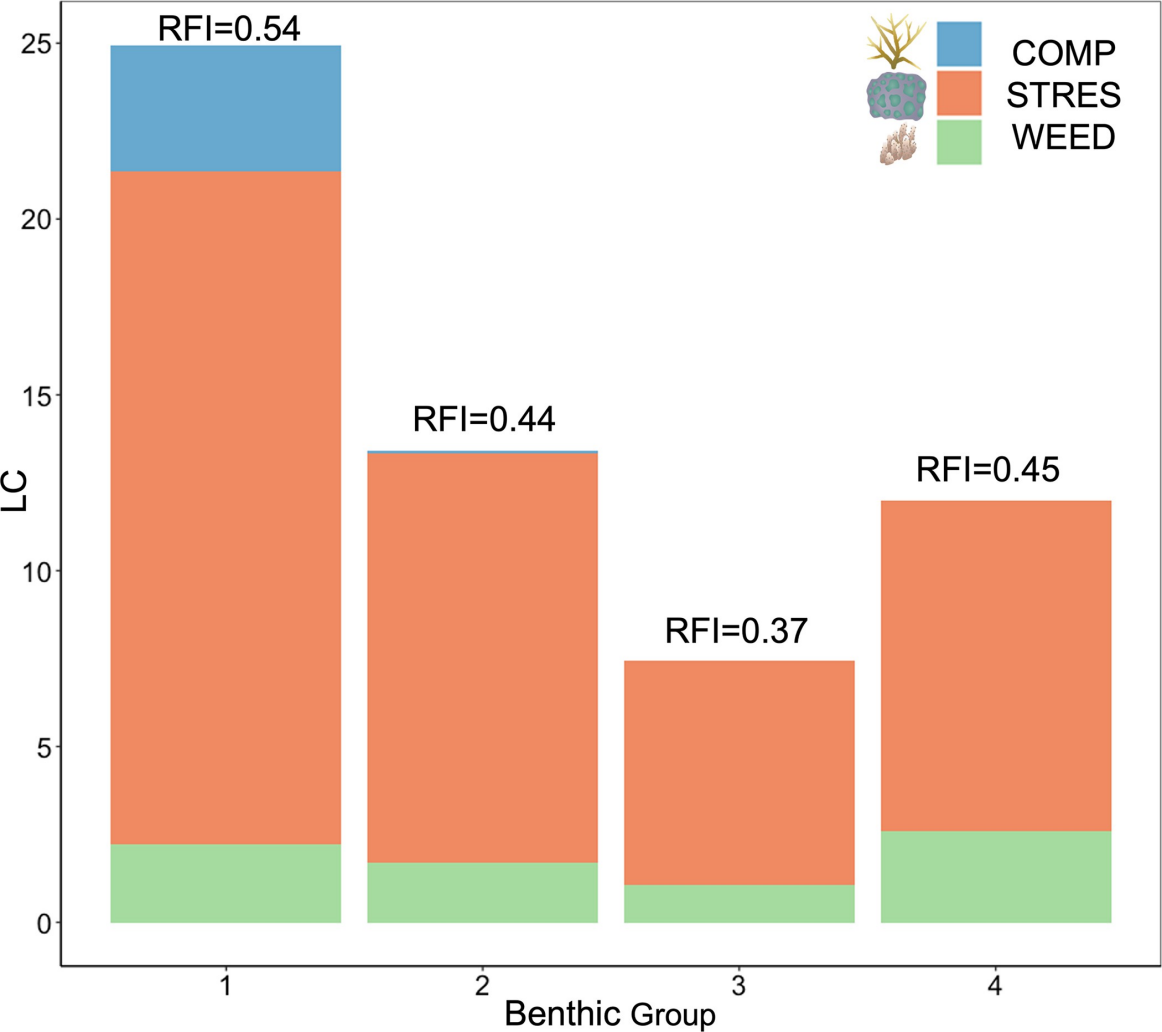
Average coral cover of all coral life-history strategies in each benthic group. COMP is competitive, STRES is stress-tolerant, and WEED is a weedy life-history strategy. RFI is the group average Reef functional index.

Considering the presence of different coral life-history strategies, all the sites in Alacranes Reef show a similar structure, dominated by stress-tolerant species mainly from the genera *Orbicella*, *Montastraea*, *Pseudodiploria*, and *Siderastrea*, followed by weedy species from the genera *Agaricia* and *Porites* (Fig 5 and S1 Table). Yet, the total abundance of each coral life-history strategy differentiates the groups. The sites in group 1 had the highest average reef functional index (RFI) values, as they had the highest coral cover and presence of all the coral life-history strategies, including the competitive acroporids (Fig 5). Groups 2 and 4 had similar RFI averages, although higher for group 4, because of the abundance of structurally complex species from the genus *Orbicella* (Fig 5 and S1 Table). Group 3 had the lowest RFI average because of its lowest hard coral cover (Fig 5).

**Fig 6:**
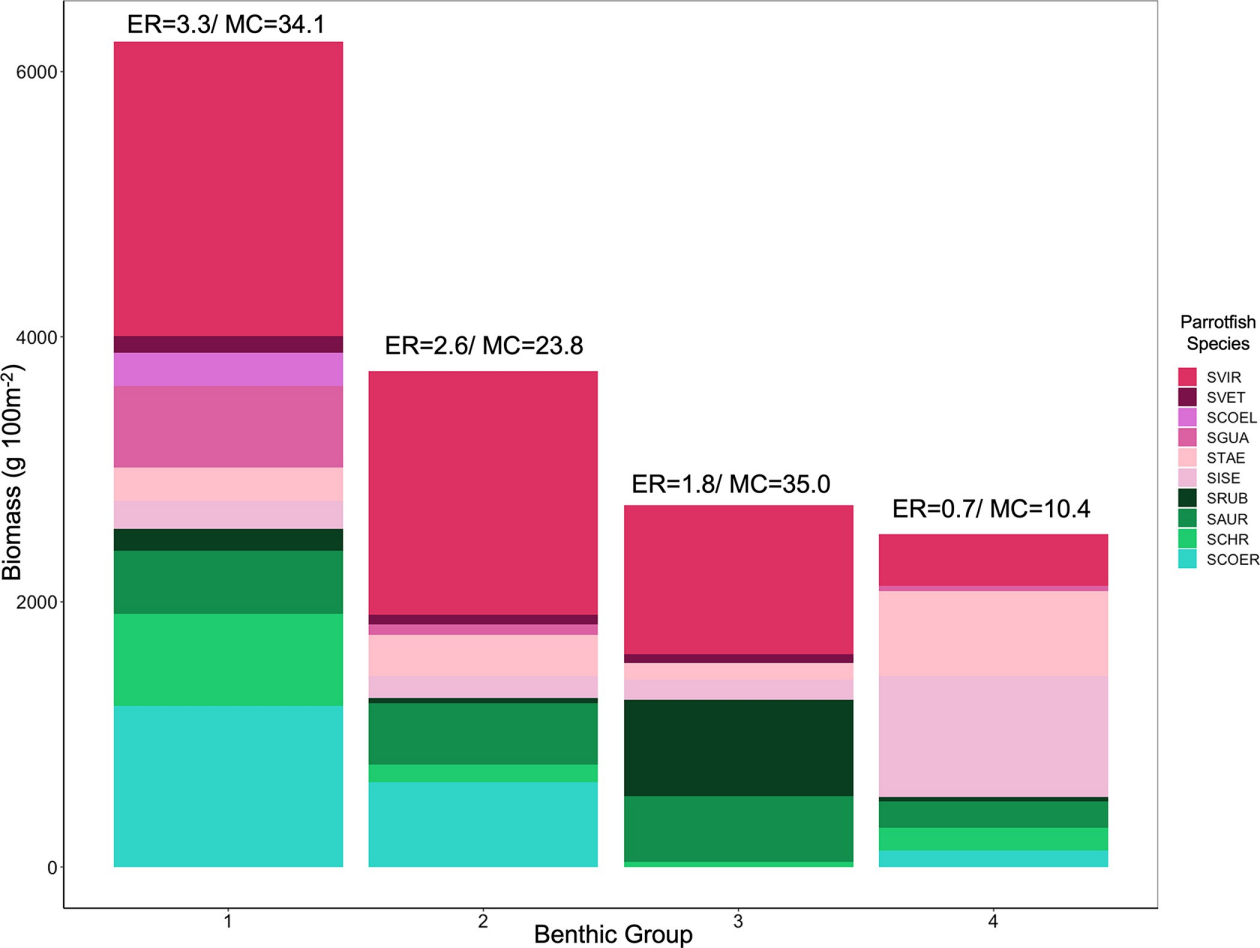
Average biomass of ten parrotfish species in each benthic group. The SVIR is *Sparisoma viride*, *the* SVET is *Scarus vetula*, the SCOEL is *Scarus coelestinus*, the SGUA is *Scarus guacamaia*, the STAE is *Scarus taeniopterus*, the SISE is *Scarus iseri*, the SRUB is *Sparisoma rubripinne*, the SAUR is *Sparisoma aurofrenatum*, *the* SCHR is *Sparisoma chrysopterum*, *a*nd the SCOER *is Scarus coeruleus*. ER is the group average erosion rate potential, and MC is the group average macroalgae consumption potential. Species with higher values of bioerosion are represented by pink hues, species with higher values of macroalgae consumption are represented by green hues, and species with low values of both functions are represented by turquoise.

For parrotfish assemblages, group 1 was characterized by a richer and more abundant assemblage in which species such as *Sparisoma viride*, *Scarus guacamaia*, *Sp*. *chrysopterum*, and *Sp*. *aurofrenatum* contributed the most to the total biomass, resulting in high values for both parrotfish functions: bioerosion and macroalgae consumption (Fig 6). Group 2 has a species-diverse assemblage but lower total biomass, which yielded lower values for both parrotfish functions than group 1 (Fig 6). Groups 3 and 4 showed less diverse and abundant parrotfish assemblages (Fig 6), dominated by *Sp*. *viride* in the former group and by small species such as *Sc*. *taeniopterus*, and *Sc*. *iseri* in the latter. Despite the relatively low overall biomass, group 3 had the highest biomass of the two species with the strongest macroalgae removal rates (i.e. *Sp*. *aurofrenatum* and *Sp*. *rubripinne*), which explains why group 3 had an elevated macroalgae consumption potential (Figs 3 and 6). *Sp*. *aurofrenatum* showed similar biomass across different benthic conditions (i.e., groups 1, 2, and 3), despite the described benthic differences.

**Fig 7:**
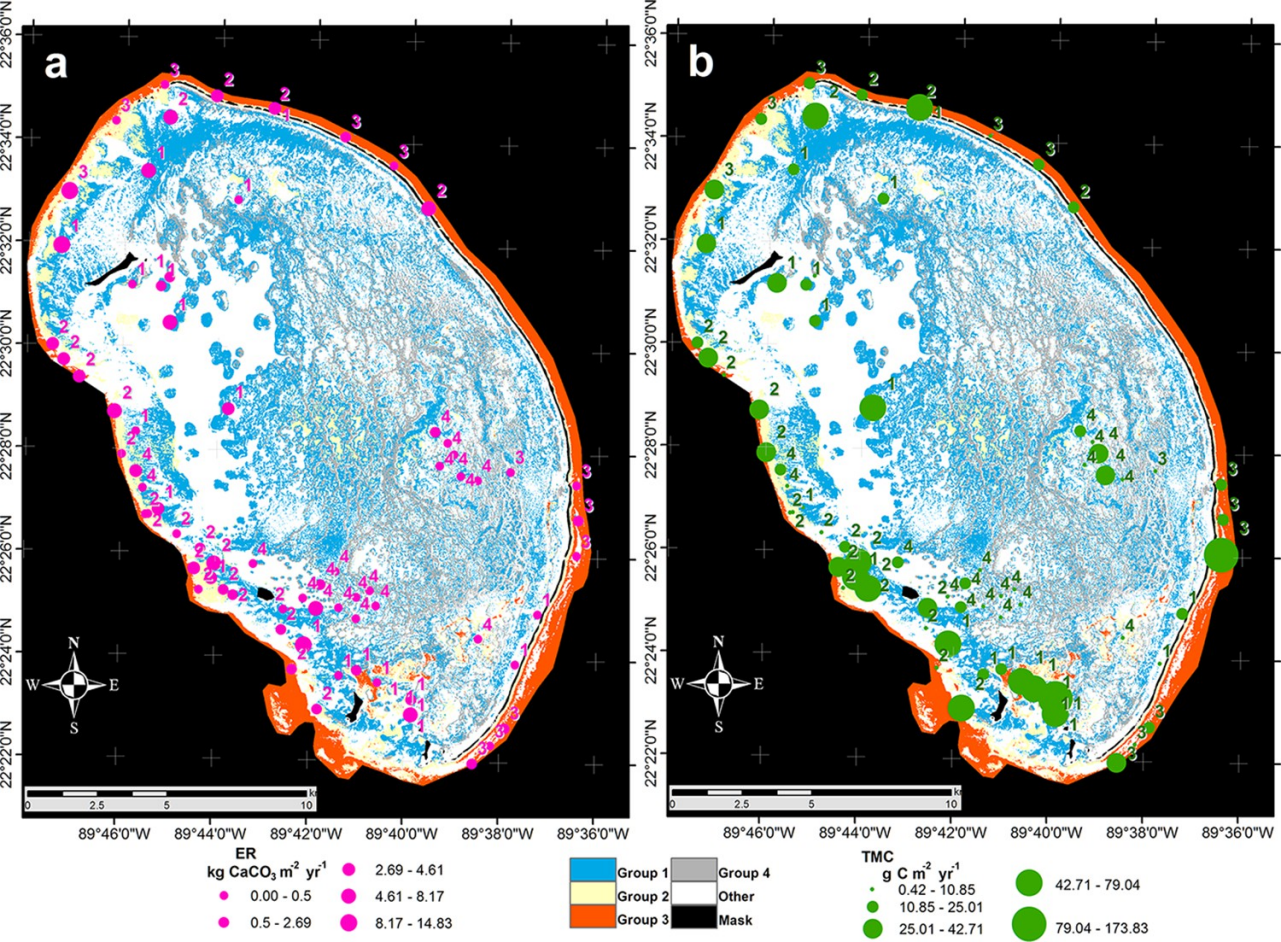
Habitat map where symbols represent the magnitude of each parrotfish function at the surveyed sites: (a) bioerosion potential in pink and (b) macroalgae consumption potential in green. The colors represent different benthic groups: blue for group 1, yellow for group 2, red for group 3, and gray for group 4. The accuracy assessment of the classified image was carried out using 25% (88) of the 350 training fields with an overall accuracy of 86.1% and a Kappa Coefficient of 0.85. Image courtesy of Planet Team (2022), Planet Application Program Interface: In Space for Life on Earth. San Francisco, CA. https://api.planet.com.

The spatial patterns in the reef platform of Alacranes, represented in the habitat map (5 classes, Overall Accuracy is 86.1, and Kappa Coefficient is 0.85), showed that the sites on the inner west reef platform were mainly characterized by high coral cover (group 1) and high values of both parrotfish functions (Fig 7). On the western border of the reef platform, most of the sites have a medium coral cover (group 2), with medium values of both parrotfish functions (Fig 7). The northern and eastern borders of the reef platform are mostly characterized by deeper sites dominated by fleshy macroalgae (group 3), depicted by high and medium values of macroalgae consumption potential (Fig 7). Finally, a high proportion of reef sites dominated by cyanobacteria (group 4) are in the southern portion towards the center of the reef platform, where erosion rates and macroalgae consumption potential are low (Fig 7).

**Fig 8:**
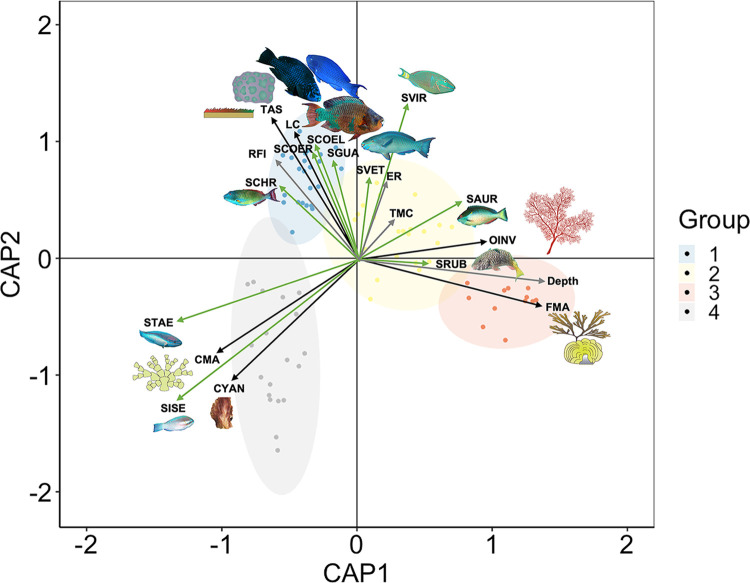
Canonical analysis of principal coordinates (CAP) of benthic conditions of the Alacranes Reef. The colors of the sites represent the benthic conditions: blue indicates benthic group 1, yellow indicates benthic group 2, red indicates benthic group 3, and gray indicates benthic group 4. Parrotfish biomass is plotted with green arrows: SCOEL is *Scarus coelestinus*, SCOER *is S*. *coeruleus*, SGUA is *S*. *guacamaia*, SISE is *S*. *iseri*, STAE is *S*. *taeniopterus*, SVET is *S*. *vetula*, SAUR is *Sparisoma aurofrenatum*, SCHR is *Sp*. *chrysopterum*, SRUB is *Sp*. *rubripinne*, and SVIR is *Sp*. *viride*. Benthic variables are plotted in black: LC is live coral, TAS is turf algae with sediments, OINV is soft coral, FMA is fleshy macroalgae, CMA is calcareous macroalgae, and CYAN is cyanobacteria. Other variables are plotted as gray arrows: the reef functional index (RFI), depth, total macroalgae consumption (TMC), and erosion rate (ER).

According to our integrative analysis (Fig 8), a gradient of depth and benthic dominance drive the benthic associations of groups 1, 2, and 3. Where group 1 is characterized by a greater hard coral cover, thus greater RFI values and is mainly associated with the large-size parrotfish species *S*. *guacamaia* (SGUA), *S*. *coeruleus* (SCOER), and *S*. *coelestinus* (SCOEL); group 2 has intermediate hard coral and fleshy macroalgae and is associated with both large and medium-size parrotfish species *Sp*. *viride* (SVIR), *S*. *vetula* (SVET), and *Sp*. *aurofrenatum* (SAUR), and group 3 is characterized by a high fleshy macroalgae cover and is mainly associated with medium-size parrotfish species *Sp*. *aurofrenatum* (SAUR) and *Sp*. *rubripinne* (SRUB). Finally, group 4 was mostly associated with cyanobacteria dominance and small parrotfish species *S*. *taeniopterus* (STAE) and *S*. *iseri* (SISE).

**Fig 9:**
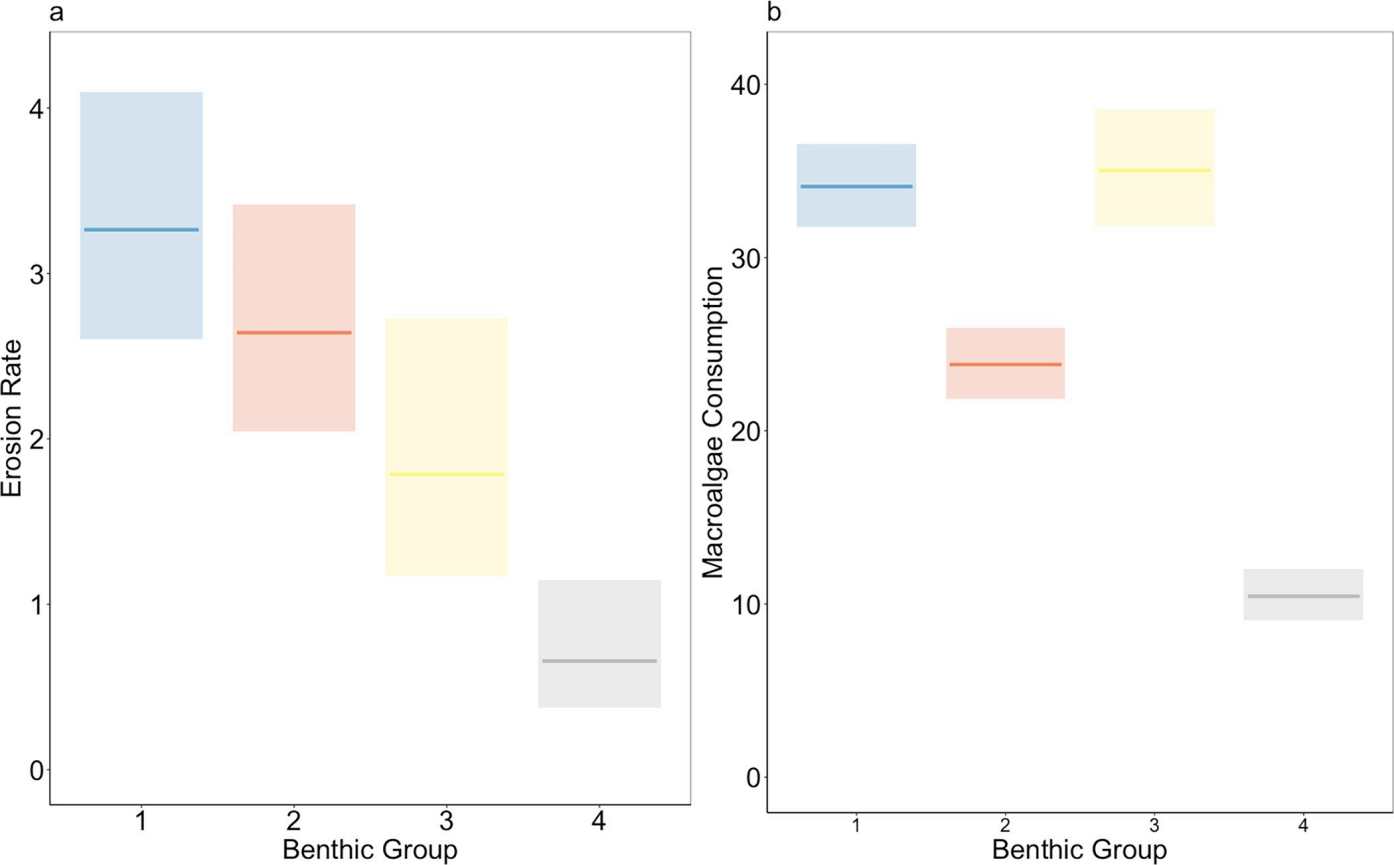
Generalized linear models of (a) the erosion rate potential and (b) the macroalgae consumption potential as a function of the benthic group (1–4). The shadowed area represents the 95% confidence interval. Blue indicates benthic group 1, yellow indicates benthic group 2, red indicates benthic group 3, and gray indicates benthic group 4.

The function of bioerosion responded to the depth and benthic dominance gradient (Fig 9), decreasing on average from group 1 (shallow sites with high coral cover) to group 3 (deep sites with low coral cover) while, the macroalgae consumption function has a more complex response to changes in benthic dominance, since it has greater values in sites with both higher coral cover (group 1) and fleshy macroalgae cover (group 3) and intermediate values in sites with medium cover of both hard coral and fleshy macroalgae (group 2). For both parrotfish functions, the lowest values occurred at the cyanobacteria-dominated sites (Fig 9).

## 4. Discussion

Our findings provide new insights into parrotfish functional variability through different reef habitats. We showed that the presence and size-weighted abundance of parrotfish species -hence their potential specific functions- are strongly influenced by benthic habitat characteristics. In Alacranes Reef, both, bioerosion and herbivory potential are greatest at structurally complex sites, driven by a high cover of massive boulders, brain, and branching coral species. Moreover, macroalgae consumption potential is also high at deep sites with low structural complexity dominated by fleshy macroalgae. Interestingly, both functions are highly diminished in shallow and reticulated inner reefs dominated by turf algae with sediments and cyanobacteria, suggesting that even herbivory potential can be depleted in unfavorable benthic conditions to sustain parrotfish assemblages.

**Fig 10:**
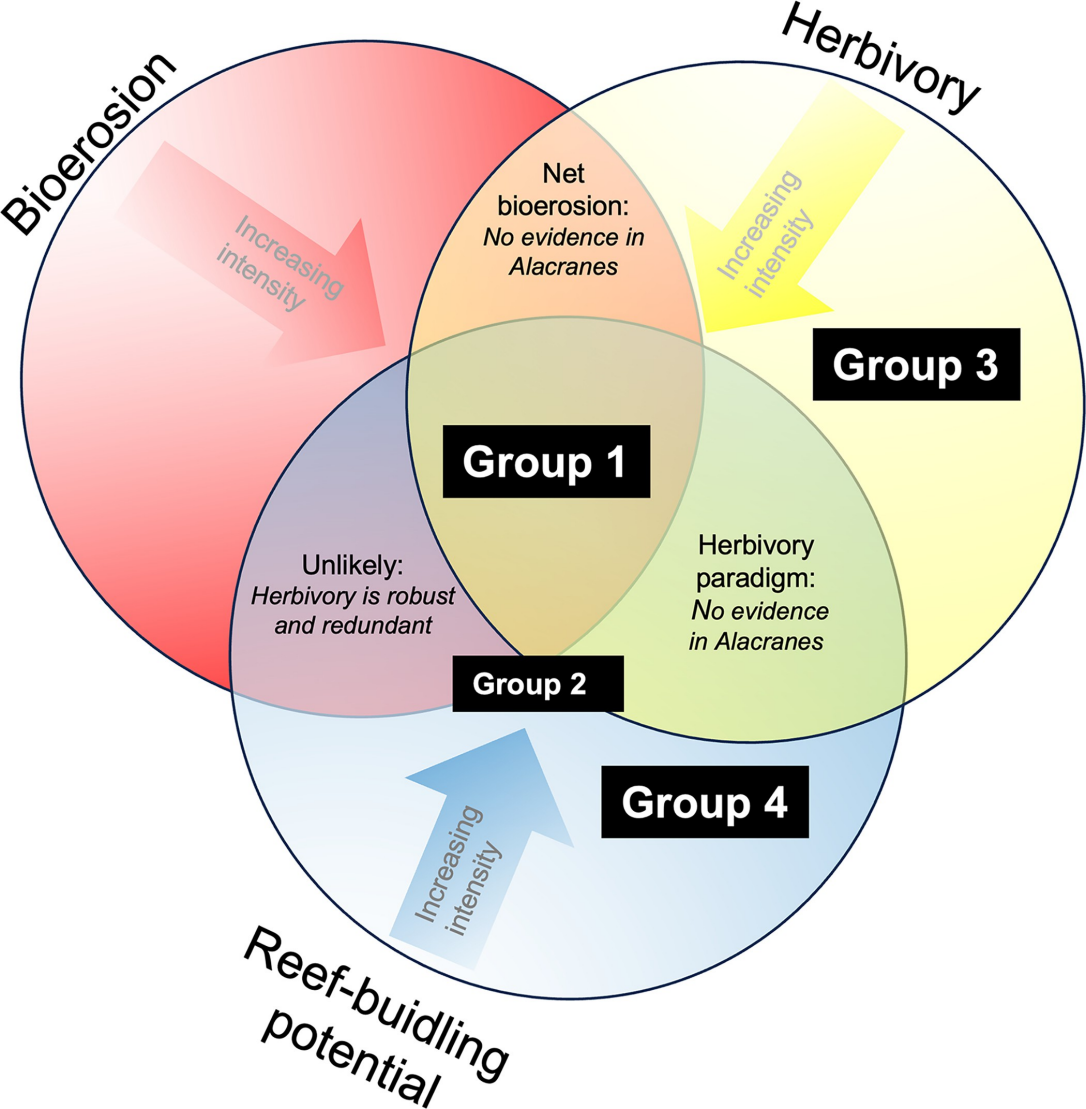
Three main reef processes: reef-building potential, bioerosion, and herbivory, as well as their interactions, were examined in the Alacranes Reef System. The scenario where all the processes interact is given by reefs where a high reef-building potential -higher than bioerosion- maintains a rich and abundant assemblage of parrotfish and their specific functions, and herbivory keeps macroalgae at low levels. The scenario where reef-building potential and herbivory interact represents the herbivory paradigm: herbivores can maintain low macroalgae levels and favor coral growth [41]. This scenario is the dominant paradigm for former Caribbean reefs, but we did not find evidence of it in the Alacranes reef. The scenario where bioerosion and herbivory interact represents potential net bioerosion because the reef-building potential is low. The interaction between reef-building potential and bioerosion is unlikely, as herbivory is a robust and redundant function. Labels for benthic groups from 1 to 4 were placed in the figure depending on their process level.

Using the Alacranes Reef seascape heterogeneity as an ecological model [29], we have approximated three main processes interacting in reefs (Fig 10): reef-building potential (driven by scleractinian cover and complexity), bioerosion (driven by large-sized parrotfish), and herbivory (driven by medium-sized parrotfish). The interactions of these three main processes are approached by three potential scenarios. Firstly, the scenario in which all three processes interact, where there is a high reef-building potential maintaining a rich and abundant assemblage of parrotfish and their specific functions, and herbivory keeps macroalgae at low levels. Secondly, the scenario in which reef-building potential and herbivory interact represents the herbivory paradigm, where herbivores can maintain low macroalgae levels and favor coral growth. Finally, the scenario where bioerosion and herbivory interact is associated with net bioerosion. These three processes and their interactions are key, as they build and shape the structure and functioning of coral reefs, thereby influencing reef-associated biodiversity and ecosystem services [23].

In particular, the herbivory potential of parrotfish is high in habitats with contrasting levels of fleshy macroalgae: low cover (~2% in group 1) and high cover (~43% in group 3), and it is not directly correlated with the total parrotfish biomass. These results agree with previous studies, which have not found a direct link between parrotfish and macroalgae abundance [8, 9]. Here, we present evidence that herbivory potential is highly dependent on the biomass of medium-sized efficient herbivores (*Sp*. *rubripinne*, *Sparisoma aurofrenatum*, and *Sp*. *chrysopterum*), which can also be associated with a range of different reef habitats (i.e., high and low structural complexity). As underlined in our hypothesis, the herbivory potential is more robust to changes in benthic components, as it is driven by a more redundant and generalist parrotfish assemblage, characterized by a high abundance of *Sparisoma aurofrenatum* and *Sp*. *rubripinne*.

Yet, in Alacranes Reef, we did not find evidence of three simultaneous processes that could support the herbivory paradigm: high reef building and herbivory potential but low bioerosion potential (Fig 10), which is in resonance with other studies highlighting that empirical evidence of this paradigm is still insufficient [42]. Furthermore, our findings indicate that in some contexts, increasing parrotfish herbivory potential might not be enough to regulate and reverse macroalgae domination (group 3). For instance, deep sites in our study system are naturally characterized by relatively high levels of sediments and nutrients and were largely dominated by fleshy macroalgae. Although herbivory was also high in those sites (Figs 6 and 8), we did not find evidence suggesting that parrotfishes were controlling or reducing the amount of fleshy macroalgae on those sites. Contrastingly, in shallower sites, the fleshy macroalgae cover tended to be lower. But it is likely, that in those sites, other key herbivores such as sea urchins, which are abundant in shallow sites of Alacranes (pers. obs. LAF) are contributing to macroalgae control. Healthy densities of sea urchins, such as *Diadema antillarum*, have been associated with low values of macroalgae and coral cover recovery in reefs [43] but are limited to the shallow distribution range of this species [44].

Conversely, as hypothesized, our analysis indicated that bioerosion potential is more sensitive to changes in benthic habitat conditions, as it is highly dependent on large parrotfish species (*Sp*. *viride*, *Sc*. *vetula*, *Sc*. *coelestinus*, and *Sc*. *guacamaia)*, which are mainly associated with structurally complex sites [15–22]. The sites where all three processes are high (group 1) correspond to reefs dominated by structurally complex *Orbicella* and *Acropora spp*., associated with high reef-building potential, maintaining a rich and abundant assemblage of parrotfish and their specific functions, where larger parrotfish drive the total biomass. These findings follow the trends where larger fish biomass (including the Scaridae family) is correlated to big and structurally complex coral colonies [10, 45]. Remarkably, these results contrast with the trend in contemporary Caribbean reefs, mainly characterized by low structural complexity [14] and low biomass of large parrotfish [6] where declining trends in parrotfish biomass and modal length have been described [46, 47]. Indeed, in Alacranes Reef, persistent favorable benthic conditions (high structural complexity) might create positive feedbacks that sustain highly dynamic reefs and maintain the carbonate accretion balance [48].

Nonetheless, our findings also revealed some unfavorable benthic conditions, characterized by high levels of sedimentation or cyanobacteria overgrowth on dead coral structures, that cannot maintain high values of parrotfish functions, despite relatively high structural complexity (Figs 6 and 10). Inner reticular complex reefs, which are dominated by turf algae and sediments (group 2) and mostly by low-palatability cyanobacteria and other genera of calcareous macroalgae (group 4), host a less abundant and diverse parrotfish assemblage. In the latter, we even found the lowest total parrotfish biomass, which was linked to the quasi-absence of large parrotfish and the dominance of habitat generalist and sympatric small parrotfish (*S*. *iseri* and *S*. *taeniopterus*), sustaining low bioerosion and macroalgae grazing potential. It has been previously reported that scarids (particularly large-sized) preferably target short turf with low sediment loads, likely due to the higher quality of this food resource [49, 50]. Whereas, both small-sized parrotfish species are known to graze on cyanobacterial mats [51, 52], confirming their habitat use and separation from other parrotfish species assemblages. These findings are relevant in the context of the current coral reef degradation trend in the Wider Caribbean, where reefs under increased eutrophication and warmer waters are shifting to cyanobacteria dominance [51, 53, 54], leading to a possible curtailing of the rich and abundant parrotfish assemblages.

In most Caribbean reefs, acute stressors such as bleaching and disease outbreaks have induced high coral mortality in the last four decades, reducing reef-building capacity and inducing net bioerosional states [55]. At the time of our study, we did not find evidence of sites in the Alacranes reef with simultaneous low reef-building and high bioerosion potential, but we collected our data before the unprecedented mass bleaching event that affected the Wider Caribbean in 2023. In the current context, several studies in the Caribbean have proven that in reefs that experience high coral mortality, bioerosion can strongly influence carbonate budget dynamics, compromising the remaining coral skeleton structures, and thus the reef framework, further accentuating reef degradation [56–59]. Therefore, in the context of reef conditions and degradation, the benefit of increased macroalgae removal function may be outweighed by higher rates of bioerosion and loss of reef structure [10, 47, 55].

All these findings have potential implications for coral reefs and herbivores management in the Wider Caribbean, stressing the complexity of interactions between reef processes, considering reciprocal benthic-fish interactions [5], as well as the importance of nuancing between parrotfish species to account for their specific roles and functions [10, 35]. For instance, to implement a management strategy aimed at increasing macroalgae consumption by protecting overall parrotfish, it would be advantageous to identify if fishing is a factor of change in parrotfish populations, as the species that will potentially benefit from fishing protection are the large parrotfish which are the most efficient bioeroders, but also to consider the reef’s condition, assessing the reef-building potential of the targeted sites [10, 42]. Moreover, several studies in the Caribbean have demonstrated that eutrophication is the main driver of macroalgae phase shifts [60, 61], and there is no evidence that this regime can be reversed by herbivore abundance [9]. To enhance reef resilience in the Wider Caribbean, there is an urgent need to focus on improving coastal management, particularly sewage treatment, which can improve local conditions and strengthen the capacity of coral reefs to resist and recover from bleaching and disease events. In parallel, it is key to advocate for the reduction of greenhouse gas emissions from local to global scales.

## 5. Conclusion

We provide new evidence that the bioerosion potential of parrotfish is highly sensitive to benthic condition changes, as it is highly dependent on large-sized parrotfish abundance and species assemblage composition. However, the herbivory potential of parrotfish is more robust to parrotfish assemblage variation through benthic conditions. Moreover, enhancing parrotfish herbivory potential, which is a common management approach to promote resilience, might not be strong enough to reverse coral-algae phase shifts. In contrast, the erosion rates of parrotfish species at degraded sites might contribute to reef flattening and further loss of reef functions, as degraded reefs are not able to cope with bioerosion.

Under increased eutrophication and warmer waters, there is a current trend of reefs shifting to cyanobacteria dominance leading to a possible curtailing of the rich and abundant parrotfish assemblages. Although there is some grazing by small-sized parrotfish species in cyanobacterial-dominated sites, this process can be considered a parrotfish assemblage adaptation to current conditions rather than hope for its use as a management tool. These findings enhance our understanding of the complex interactions between reef processes and the importance of considering this complexity in management actions to build resilience in Wider Caribbean coral reefs.

## Supporting information

S1 Table Coral species average cover per benthic group. (DOCX)

S1 Dataset Benthic cover percentage, herbivore fish biomass and ecological indicators per sites in Alacranes reef. (ZIP)
