# Supplementary material for: Strong linkage between parrotfish functions and habitat characteristics
Source: PLoS One. 2024 Dec 11;19(12):e0315179. doi: 10.1371/journal.pone.0315179 (PMC11633996; doi:10.1371/journal.pone.0315179)
Supplement: S1 Table — (DOCX) [file pone.0315179.s001.docx]

**Supporting Information**

**S1 Table.** Coral species average cover per benthic group.

| Life history strategy | Coral Species | Group 1 (%) | Group 2 (%) | Group 3 (%) | Group 4 (%) |
| --- | --- | --- | --- | --- | --- |
| Competitive | *Acropora cervicornis* | 1.59 | 0.00 | 0.00 | 0.00 |
|  | *Acropora palmata* | 1.00 | 0.05 | 0.00 | 0.00 |
|  | *Acropora prolifera* | 0.99 | 0.00 | 0.00 | 0.00 |
| Stress-tolerant | *Colpophyllia natans* | 0.58 | 0.14 | 0.00 | 0.03 |
|  | *Diploria labyrinthiformis* | 0.11 | 0.18 | 0.00 | 0.07 |
|  | *Dichocoenia stokesi* | 0.00 | 0.00 | 0.02 | 0.01 |
|  | *Eusmilia fastigiata* | 0.00 | 0.01 | 0.00 | 0.00 |
|  | *Meandrina meandrites* | 0.04 | 0.00 | 0.03 | 0.00 |
|  | *Montastraea cavernosa* | 0.87 | 3.50 | 1.23 | 0.25 |
|  | *Orbicella annularis* | 12.87 | 1.85 | 0.43 | 6.28 |
|  | *Orbicella faveolata* | 0.65 | 0.10 | 0.03 | 0.32 |
|  | *Orbicella franksi* | 0.33 | 1.90 | 0.48 | 0.62 |
|  | *Pseudodiploria clivosa* | 0.11 | 0.10 | 0.00 | 0.16 |
|  | *Pseudodiploria strigosa* | 2.25 | 1.68 | 1.84 | 1.28 |
|  | *Stephanocoenia intersepta* | 0.02 | 0.31 | 0.21 | 0.05 |
|  | *Siderastrea siderea* | 1.31 | 1.90 | 2.13 | 0.35 |
| Weedy | *Agaricia agaricites* | 0.07 | 0.16 | 0.14 | 0.07 |
|  | *Agaricia lamarcki* | 0.00 | 0.04 | 0.00 | 0.00 |
|  | *Agaricia tenuifolia* | 0.01 | 0.00 | 0.00 | 0.00 |
|  | *Helioseris cucullata* | 0.00 | 0.00 | 0.05 | 0.00 |
|  | *Manicina areolata* | 0.00 | 0.00 | 0.00 | 0.00 |
|  | *Mycetophyllia danaana* | 0.00 | 0.01 | 0.00 | 0.00 |
|  | *Mycetophyllia lamarckiana* | 0.00 | 0.01 | 0.00 | 0.00 |
|  | *Porites astreoides* | 1.85 | 1.30 | 0.63 | 1.60 |
|  | *Porites furcata* | 0.06 | 0.01 | 0.03 | 0.03 |
|  | *Porites porites* | 0.16 | 0.12 | 0.21 | 0.79 |
|  | *Siderastrea radians* | 0.08 | 0.03 | 0.00 | 0.09 |
|  | **Total Average** | 24.95 | 13.40 | 7.46 | 12.01 |
